# Supplementary material for: A Likelihood Approach for Real-Time Calibration of Stochastic Compartmental Epidemic Models
Source: PLoS Comput Biol. 2017 Jan 17;13(1):e1005257. doi: 10.1371/journal.pcbi.1005257 (PMC5240920; doi:10.1371/journal.pcbi.1005257)
Supplement: S1 File — (TAR.GZ) [file pcbi.1005257.s014.tar.gz › HSPH_Online-SI-Revision/MSS10/n1-extreme/n1-extreme_NEW_table4.pdf]

1	R0	{2.95019, 14.2854, 0.1}	{2.53075, 11.6597, 0.}	{{2.53075, 11.6597, 0.}, {2.53075, 11.6597, 0.}}	{14.2174, 18.3802, 100.}	inf	101.	76.6366	{71.3377, 81.3656}	24.1222	0.120548	newly	40.	37.6	{31., 44.}	11.6	0.0538247	Reff	0.31272	0.329917	{0.314648, 0.343039}	5.63438	ar	924.	898.34	{886., 909.}	2.77706
2	R0	{2.67565, 16.946, 0.1}	{2.54559, 16.3017, 0.}	{{2.48143, 15.7026, 0.}, {2.93978, 19.9817, 0.}}	{7.62471, 8.81818, 100.}	inf	81.	63.2981	{56.4258, 76.5184}	22.1972	0.111122	newly	24.	23.16	{18., 28.}	14.	0.0638337	Reff	0.294322	0.324042	{0.312763, 0.335397}	10.0981	ar	926.	898.77	{886., 914.}	2.9406
3	R0	{2.40066, 16.6543, 0.1}	{2.21928, 14.4774, 0.48}	{{2.11132, 12.644, 0.}, {2.45321, 19.391, 2.}}	{9.07694, 20.9587, 532.}	inf	164.	118.272	{97.8351, 151.516}	27.9944	0.149453	newly	51.	50.47	{43., 59.}	10.2941	0.0452699	Reff	0.40091	0.468667	{0.440312, 0.489012}	16.939	ar	916.	861.37	{826., 904.}	5.99017
4	R0	{1.9294, 15.4361, 0.1}	{1.76248, 10.4932, 0.02}	{{1.67732, 9.44073, 0.}, {1.88607, 11.9453, 0.}}	{8.99511, 32.5499, 118.}	inf	105.	70.8086	{45.3559, 101.703}	34.8557	0.204496	newly	40.	41.2	{28., 54.}	21.15	0.0903862	Reff	0.677219	0.676052	{0.660269, 0.692625}	1.5788	ar	796.	711.85	{652., 781.}	10.8455
5	R0	{2.92859, 19.5393, 0.1}	{1.87099, 6.9108, 2.64}	{{1.82266, 3.08261, 0.}, {1.9862, 8.17412, 4.}}	{36.1129, 64.6313, 2584.}	inf	114.	9.62268	{4.48789, 14.3961}	91.559	1.1141	newly	40.	7.55	{3., 12.}	81.125	0.775999	Reff	0.351431	0.419864	{0.400263, 0.44744}	19.4727	ar	927.	782.62	{772., 793.}	15.575
6	R0	{1.79622, 12.3148, 0.1}	{1.65456, 3.37285, 0.}	{{1.65456, 3.37285, 0.}, {1.65456, 3.37285, 0.}}	{7.88654, 72.6114, 100.}	inf	56.	15.1803	{4.85701, 27.7189}	72.8924	0.646169	newly	31.	22.46	{7., 40.}	43.6774	0.282659	Reff	0.661009	0.676417	{0.655007, 0.693052}	2.81716	ar	734.	618.97	{585., 657.}	15.6717
7	R0	{2.45855, 17.1776, 1.}	{2.13228, 14.7292, 2.97}	{{2.12134, 14.6763, 3.}, {2.12134, 14.6763, 3.}}	{13.598, 14.5812, 197.}	inf	121.	81.1409	{73.1534, 88.1854}	34.0104	0.181154	newly	52.	33.73	{26., 41.}	35.1346	0.19371	Reff	0.486793	0.506328	{0.489423, 0.521446}	4.7435	ar	871.	823.7	{799., 845.}	5.64638
8	R0	{2.20009, 15.519, 2.}	{2.59755, 18.2464, 0.}	{{2.59755, 18.2464, 0.}, {2.59755, 18.2464, 0.}}	{18.0657, 17.5749, 100.}	inf	65.	149.049	{143.099, 154.53}	129.306	0.360204	newly	25.	50.19	{42., 58.}	100.76	0.299647	Reff	0.448818	0.31158	{0.296811, 0.326577}	30.5778	ar	839.	927.97	{919., 938.}	10.6043
9	R0	{2.06493, 9.6414, 1.}	{2.02203, 11.0282, 1.31}	{{1.96658, 10.2409, 1.}, {2.0072, 12.2033, 3.}}	{4.83098, 14.3837, 31.}	inf	54.	75.2539	{59.1324, 87.6147}	39.5099	0.128984	newly	29.	39.94	{31., 48.}	38.4828	0.133924	Reff	0.580245	0.522565	{0.499194, 0.546729}	9.94072	ar	777.	801.35	{772., 829.}	3.42471
10	R0	{2.60775, 19.6936, 1.}	{2.27582, 18.9086, 0.}	{{2.27582, 18.9086, 0.}, {2.27582, 18.9086, 0.}}	{12.7287, 3.98604, 100.}	inf	139.	149.674	{142.414, 158.545}	7.77812	0.0322427	newly	45.	48.91	{41., 58.}	13.4	0.0540631	Reff	0.47461	0.389906	{0.369541, 0.405303}	17.8471	ar	889.	895.2	{880., 909.}	1.15186
11	R0	{1.99168, 8.13555, 1.}	{1.98235, 10.6306, 1.26}	{{1.96658, 10.2409, 1.}, {2.0072, 10.6431, 3.}}	{2.59948, 31.4961, 26.}	inf	45.	62.9714	{50.5236, 74.677}	44.1454	0.156899	newly	31.	35.74	{26., 43.}	21.8065	0.0864335	Reff	0.579579	0.540167	{0.52431, 0.555686}	6.80017	ar	772.	783.06	{755., 807.}	2.73834
12	R0	{1.79304, 9.18941, 0.1}	{1.70464, 6.33701, 1.89}	{{1.59034, 5.09935, 0.}, {1.75445, 7.55154, 3.}}	{5.91928, 32.0958, 1820.}	inf	52.	39.5349	{20.1702, 53.6777}	32.2623	0.195878	newly	36.	34.	{20., 46.}	22.7222	0.124297	Reff	0.627564	0.616672	{0.600335, 0.631591}	2.36089	ar	725.	687.8	{648., 724.}	5.87586
13	R0	{1.84016, 18.3431, 1.}	{1.41667, 6.48127, 1.17}	{{1.38815, 5.7404, 0.}, {1.44485, 7.81552, 3.}}	{23.0136, 64.6665, 95.}	inf	88.	20.9625	{9.43514, 29.8338}	76.1789	0.66285	newly	32.	19.91	{8., 30.}	38.7188	0.25886	Reff	0.875916	0.769247	{0.753511, 0.783139}	12.178	ar	762.	511.34	{461., 558.}	32.895
14	R0	{1.94221, 9.85971, 1.}	{2.0968, 12.1899, 1.}	{{2.0968, 12.1899, 1.}, {2.0968, 12.1899, 1.}}	{7.95973, 23.6338, 0.}	inf	31.	46.4314	{41.3059, 51.5979}	49.7788	0.173903	newly	20.	22.63	{18., 27.}	19.45	0.0771687	Reff	0.42146	0.42265	{0.41175, 0.433258}	1.58861	ar	805.	826.15	{814., 841.}	2.64969
15	R0	{1.78578, 12.0541, 0.1}	{2.2194, 19.1478, 0.}	{{2.2194, 19.1478, 0.}, {2.2194, 19.1478, 0.}}	{24.2819, 58.8486, 100.}	inf	61.	101.753	{94.0962, 109.332}	66.809	0.221417	newly	32.	33.95	{28., 41.}	13.8438	0.0571261	Reff	0.558949	0.604224	{0.587404, 0.621188}	8.10001	ar	732.	824.9	{804., 844.}	12.6913
16	R0	{1.94463, 15.6503, 2.}	{1.61502, 7.56197, 1.44}	{{1.58886, 7.29751, 1.}, {1.70777, 8.49961, 3.}}	{16.9495, 51.6816, 50.}	inf	82.	28.2484	{14.0734, 52.6225}	65.5507	0.513193	newly	29.	21.53	{12., 37.}	38.0345	0.220734	Reff	0.663119	0.636911	{0.624104, 0.649729}	3.95227	ar	757.	649.22	{611., 697.}	14.2378
17	R0	{2.08901, 16.4314, 0.1}	{1.72212, 9.88279, 0.03}	{{1.71608, 9.75208, 0.}, {1.71608, 9.75208, 0.}}	{17.5628, 40.118, 124.}	inf	116.	60.8994	{49.9029, 70.4203}	48.0966	0.290059	newly	42.	37.19	{28., 46.}	16.2619	0.0790419	Reff	0.676839	0.652283	{0.635386, 0.668304}	3.62908	ar	826.	702.81	{670., 730.}	14.914
18	R0	{2.76419, 18.56, 2.}	{2.8706, 18.4585, 1.}	{{2.86501, 18.4249, 1.}, {2.86501, 18.4249, 1.}}	{3.84949, 0.792073, 50.}	inf	153.	158.688	{153.22, 164.929}	3.96389	0.0166737	newly	51.	53.06	{45., 62.}	10.4314	0.0438985	Reff	0.351052	0.348161	{0.329679, 0.363065}	3.19504	ar	920.	934.24	{922., 945.}	1.56522
19	R0	{2.3821, 17.1399, 2.}	{2.25224, 15.2098, 2.61}	{{2.22867, 14.662, 2.}, {2.29887, 15.6411, 3.}}	{5.45144, 11.2609, 30.5}	inf	162.	115.583	{103.235, 124.683}	28.6523	0.147642	newly	73.	47.52	{39., 56.}	34.9041	0.190225	Reff	0.443071	0.523523	{0.497393, 0.556059}	18.158	ar	883.	853.14	{835., 872.}	3.42242
20	R0	{2.78314, 19.6127, 0.1}	{2.14541, 14.4943, 0.}	{{2.12155, 14.3165, 0.}, {2.2277, 15.0861, 0.}}	{22.9139, 26.0973, 100.}	inf	71.	16.7942	{3.21291, 57.7138}	77.101	0.883477	newly	24.	6.8	{1., 22.}	73.75	0.85378	Reff	0.222651	0.286744	{0.207316, 0.312795}	32.2416	ar	943.	870.62	{853., 920.}	7.71156
21	R0	{1.85124, 14.6794, 0.1}	{2.036, 18.765, 2.}	{{2.036, 18.765, 2.}, {2.036, 18.765, 2.}}	{9.98042, 27.8324, 1900.}	inf	108.	125.445	{117.429, 134.305}	16.153	0.0644491	newly	46.	45.07	{38., 52.}	9.76087	0.043405	Reff	0.794182	0.837926	{0.819609, 0.853441}	5.50808	ar	730.	784.67	{756., 814.}	7.48904
22	R0	{2.1028, 14.9852, 2.}	{1.94282, 12.1446, 2.}	{{1.94282, 12.1446, 2.}, {1.94282, 12.1446, 2.}}	{7.60815, 18.9557, 0.}	inf	84.	45.3461	{38.6559, 51.0257}	46.0165	0.270016	newly	32.	22.6	{17., 28.}	29.625	0.159393	Reff	0.471027	0.510288	{0.496825, 0.521987}	8.33516	ar	840.	778.11	{759., 796.}	7.36786
23	R0	{1.86601, 10.2157, 1.}	{1.9394, 12.1033, 2.01}	{{1.94282, 12.1446, 2.}, {1.94282, 12.1446, 2.}}	{4.13453, 19.0078, 101.}	inf	60.	81.9768	{72.5161, 91.0347}	37.0245	0.135189	newly	37.	42.59	{35., 51.}	17.6486	0.0681248	Reff	0.649371	0.632201	{0.612299, 0.651069}	2.89967	ar	747.	771.21	{742., 797.}	3.53548
24	R0	{2.34032, 10.0992, 0.1}	{2.44342, 11.4364, 0.}	{{2.22376, 9.35432, 0.}, {2.52588, 12.2207, 0.}}	{7.17089, 17.4291, 100.}	inf	58.	58.831	{35.5194, 70.8579}	20.3201	0.0951377	newly	35.	29.76	{21., 39.}	19.8286	0.102478	Reff	0.355729	0.36763	{0.351025, 0.388002}	4.12534	ar	887.	877.07	{849., 896.}	1.66967
25	R0	{1.91548, 15.3401, 1.}	{1.68737, 9.60803, 0.03}	{{1.67732, 9.44073, 0.}, {1.67732, 9.44073, 0.}}	{12.2123, 37.3666, 97.}	inf	102.	77.8128	{64.6456, 85.8965}	26.9908	0.135382	newly	45.	50.43	{42., 60.}	16.9111	0.0675902	Reff	0.739375	0.691335	{0.674456, 0.70749}	6.49745	ar	765.	703.13	{671., 737.}	8.51634
26	R0	{2.75283, 10.6721, 0.1}	{2.96508, 15.9199, 0.}	{{2.96947, 15.963, 0.}, {2.96947, 15.963, 0.}}	{7.87176, 49.1734, 100.}	inf	40.	68.2943	{65.3631, 71.6368}	70.7358	0.2317	newly	22.	25.28	{21., 30.}	17.6364	0.0682411	Reff	0.286294	0.224374	{0.21393, 0.233472}	21.6283	ar	926.	941.69	{934., 949.}	1.71382
27	R0	{2.08367, 19.7849, 1.}	{1.69866, 15.0596, 3.}	{{1.70173, 15.1079, 3.}, {1.70173, 15.1079, 3.}}	{18.4777, 23.8834, 200.}	inf	132.	75.908	{67.2398, 84.8108}	42.4939	0.247438	newly	42.	31.78	{25., 39.}	25.5714	0.136002	Reff	0.614683	0.596275	{0.581978, 0.606005}	3.75321	ar	833.	730.51	{700., 756.}	12.3037
28	R0	{2.64334, 17.1765, 0.1}	{2.84971, 19.8863, 2.}	{{2.86095, 19.9929, 2.}, {2.86095, 19.9929, 2.}}	{8.32852, 16.3618, 1900.}	inf	110.	125.011	{119.933, 130.952}	15.0326	0.061585	newly	34.	38.6	{30., 47.}	17.8235	0.0701706	Reff	0.311914	0.292892	{0.277639, 0.306566}	6.86409	ar	934.	934.67	{926., 945.}	0.716274
29	R0	{2.87008, 13.3872, 0.1}	{2.22034, 7.1404, 2.8}	{{2.20704, 6.93514, 2.}, {2.25647, 7.29612, 3.}}	{22.6383, 46.6625, 2700.}	inf	80.	9.08981	{3.33163, 18.4579}	88.6377	1.02024	newly	36.	6.6	{2., 12.}	81.6667	0.809079	Reff	0.226736	0.33277	{0.316417, 0.343979}	46.765	ar	941.	854.17	{846., 865.}	9.22742
30	R0	{2.06105, 19.6275, 1.}	{1.64375, 14.5909, 2.34}	{{1.63746, 10.1332, 0.}, {1.64552, 15.8482, 3.}}	{20.2471, 25.6608, 178.}	inf	42.	65.7161	{44.5498, 75.2222}	57.0062	0.189192	newly	18.	27.12	{22., 32.}	50.6667	0.17307	Reff	0.595643	0.436024	{0.420824, 0.466764}	26.7978	ar	747.	776.94	{750., 796.}	4.13922
31	R0	{2.30764, 13.435, 2.}	{2.58793, 15.4078, 1.}	{{2.47514, 14.5291, 1.}, {2.60786, 15.5928, 1.}}	{12.5927, 15.6828, 50.}	inf	82.	99.4659	{76.6973, 118.994}	26.0462	0.10098	newly	42.	38.69	{31., 47.}	13.7381	0.0656303	Reff	0.376145	0.374505	{0.341476, 0.411871}	5.93188	ar	877.	898.25	{873., 920.}	2.98632
32	R0	{2.30565, 11.9237, 0.1}	{2.6141, 15.6681, 1.}	{{2.47514, 14.5291, 1.}, {2.90165, 18.0404, 1.}}	{13.3781, 31.4032, 900.}	inf	65.	98.4379	{84.7081, 112.359}	51.443	0.177811	newly	34.	37.66	{30., 45.}	15.5294	0.0621678	Reff	0.378127	0.339976	{0.321649, 0.356988}	10.0894	ar	853.	908.22	{889., 928.}	6.47362
33	R0	{2.24031, 18.582, 1.}	{2.21466, 19.0909, 0.}	{{2.2194, 19.1478, 0.}, {2.2194, 19.1478, 0.}}	{1.14471, 3.2289, 100.}	inf	156.	157.589	{150.348, 166.223}	4.03415	0.0179221	newly	47.	53.34	{45., 64.}	16.3404	0.0646022	Reff	0.578	0.567611	{0.548992, 0.583967}	2.51903	ar	864.	860.92	{841., 880.}	1.50231
34	R0	{2.33306, 15.9759, 0.1}	{1.97355, 11.0919, 1.02}	{{1.87927, 9.84456, 1.}, {1.98131, 11.1772, 1.}}	{15.4532, 30.804, 920.}	inf	110.	65.2753	{44.2907, 75.1974}	41.4421	0.238665	newly	38.	34.52	{24., 43.}	15.0526	0.0742606	Reff	0.468945	0.484131	{0.467028, 0.502648}	3.83386	ar	854.	803.33	{773., 828.}	6.137
35	R0	{2.48357, 18.3573, 1.}	{2.08003, 13.1443, 2.}	{{2.08003, 13.1443, 2.}, {2.08003, 13.1443, 2.}}	{16.2482, 28.3973, 100.}	inf	120.	84.4095	{77.13, 91.5641}	29.6587	0.153937	newly	42.	40.17	{32., 47.}	10.8333	0.0497832	Reff	0.588606	0.566998	{0.55177, 0.581898}	3.77666	ar	835.	804.76	{786., 821.}	3.6479
36	R0	{2.59548, 19.9046, 0.1}	{2.30108, 15.555, 0.}	{{2.30108, 15.555, 0.}, {2.30108, 15.555, 0.}}	{11.3427, 21.852, 100.}	inf	123.	105.963	{98.5799, 112.346}	13.8511	0.0652586	newly	46.	42.44	{35., 53.}	13.6957	0.0641761	Reff	0.490546	0.474108	{0.45939, 0.490711}	3.49745	ar	863.	862.21	{844., 878.}	1.2781
37	R0	{2.29641, 16.9868, 0.1}	{1.70787, 15.5687, 1.04}	{{1.70497, 15.8627, 1.}, {1.70497, 15.8627, 1.}}	{25.6287, 8.34823, 940.}	inf	111.	29.3313	{23.2349, 34.6001}	73.5754	0.593655	newly	31.	11.53	{8., 15.}	62.8065	0.448913	Reff	0.420243	0.452089	{0.442252, 0.461425}	7.57808	ar	877.	757.62	{743., 772.}	13.6123
38	R0	{2.65743, 15.4569, 1.}	{2.56673, 16.9911, 0.}	{{2.56757, 17.005, 0.}, {2.56757, 17.005, 0.}}	{3.62545, 10.2898, 100.}	inf	85.	109.561	{103.795, 114.738}	29.6332	0.112785	newly	40.	39.05	{32., 46.}	11.675	0.0525808	Reff	0.385327	0.308711	{0.295463, 0.3217}	19.8834	ar	897.	916.55	{906., 928.}	2.25975
39	R0	{2.67953, 14.5132, 2.}	{2.95297, 18.2757, 0.}	{{2.96797, 18.4042, 0.}, {2.96797, 18.4042, 0.}}	{10.6786, 26.0872, 100.}	inf	119.	159.188	{152.887, 167.116}	33.7716	0.125745	newly	46.	53.28	{43., 63.}	17.7391	0.0691031	Reff	0.404609	0.326693	{0.304439, 0.347395}	19.2571	ar	894.	939.73	{928., 949.}	5.11521
40	R0	{1.94594, 11.928, 1.}	{2.15292, 16.7623, 3.}	{{2.14288, 16.6365, 3.}, {2.14288, 16.6365, 3.}}	{10.6366, 40.5288, 200.}	inf	60.	99.7714	{92.9934, 106.11}	66.2857	0.220136	newly	31.	37.89	{31., 46.}	23.9677	0.0901677	Reff	0.558485	0.532095	{0.51819, 0.54586}	4.73993	ar	796.	831.1	{811., 847.}	4.40955
41	R0	{1.66297, 10.0047, 0.1}	{1.66926, 12.0739, 1.}	{{1.64295, 11.759, 1.}, {1.64295, 11.759, 1.}}	{2.59289, 20.6824, 900.}	inf	53.	88.9384	{75.7445, 96.7986}	67.8083	0.21697	newly	33.	44.89	{36., 57.}	36.697	0.129615	Reff	0.640243	0.581075	{0.564587, 0.595067}	9.24163	ar	716.	740.43	{711., 765.}	3.88128
42	R0	{2.91568, 16.4453, 1.}	{2.95453, 18.3807, 1.55}	{{2.93371, 17.1903, 1.}, {2.97157, 19.3547, 2.}}	{1.33253, 11.7689, 55.}	inf	138.	125.799	{117.63, 132.451}	8.84139	0.0405974	newly	55.	42.71	{35., 51.}	22.3818	0.113671	Reff	0.297399	0.337359	{0.316374, 0.358744}	13.4363	ar	919.	932.09	{920., 945.}	1.46572
43	R0	{2.55898, 15.8889, 0.1}	{2.12424, 12.1478, 0.98}	{{2.0072, 10.6431, 1.}, {2.1245, 12.2645, 1.}}	{17.6281, 24.1786, 884.}	inf	147.	78.315	{65.0518, 85.6973}	47.9844	0.286037	newly	62.	38.83	{32., 46.}	37.371	0.208534	Reff	0.455498	0.521797	{0.506568, 0.539548}	15.1226	ar	904.	816.97	{790., 838.}	9.79535
44	R0	{2.28649, 15.9017, 0.1}	{2.43171, 15.6862, 0.21}	{{2.30907, 14.0824, 0.}, {2.49092, 17.9391, 1.}}	{6.35136, 6.7364, 268.}	inf	92.	103.31	{91.2389, 115.948}	13.1818	0.0525007	newly	43.	40.7	{33., 49.}	12.2791	0.0568032	Reff	0.400136	0.397583	{0.373839, 0.422932}	3.89378	ar	880.	884.87	{866., 903.}	1.32841
45	R0	{2.65121, 18.6339, 0.1}	{2.48232, 14.213, 0.75}	{{2.14747, 10.7247, 0.}, {2.60786, 15.5255, 1.}}	{7.12602, 23.7248, 706.}	inf	160.	118.4	{77.1485, 142.361}	25.9998	0.142047	newly	45.	50.3	{39., 59.}	16.4	0.0651537	Reff	0.371169	0.445698	{0.412267, 0.478738}	20.0794	ar	928.	883.52	{832., 913.}	4.7931
46	R0	{2.52291, 18.0251, 2.}	{2.11292, 12.9719, 0.}	{{2.11292, 12.9719, 0.}, {2.11292, 12.9719, 0.}}	{16.2507, 28.0342, 100.}	inf	131.	85.1875	{77.3237, 93.0625}	34.9714	0.188049	newly	46.	39.16	{32., 46.}	15.913	0.078132	Reff	0.431418	0.458107	{0.439636, 0.473773}	6.30822	ar	894.	839.86	{819., 856.}	6.05593
47	R0	{2.22618, 17.1225, 0.1}	{1.81589, 10.5531, 0.06}	{{1.81647, 10.5184, 0.}, {1.81647, 10.5184, 0.}}	{18.4304, 38.3672, 154.}	inf	148.	69.4583	{59.8199, 79.946}	53.0687	0.331105	newly	59.	40.8	{33., 50.}	30.9492	0.166299	Reff	0.634461	0.660161	{0.639751, 0.677624}	4.20862	ar	850.	726.29	{692., 761.}	14.5541
48	R0	{2.04405, 16.073, 1.}	{1.64404, 10.7438, 4.}	{{1.59682, 9.30931, 4.}, {1.77361, 14.2286, 4.}}	{19.5695, 33.1564, 300.}	inf	103.	62.2301	{31.3916, 115.558}	44.602	0.300124	newly	40.	33.16	{20., 51.}	32.4	0.171376	Reff	0.711329	0.636921	{0.589912, 0.669662}	10.4604	ar	779.	686.13	{611., 779.}	12.294
49	R0	{2.84396, 18.097, 1.}	{2.80792, 16.5606, 2.}	{{2.48103, 13.7658, 2.}, {2.96398, 17.7348, 2.}}	{4.34855, 8.48977, 100.}	inf	132.	110.374	{86.9611, 128.54}	16.5575	0.0863899	newly	43.	40.47	{33., 48.}	11.9302	0.0557902	Reff	0.264488	0.318446	{0.28401, 0.367886}	20.4339	ar	952.	922.07	{886., 943.}	3.14391
50	R0	{1.85341, 18.0975, 4.}	{1.99485, 17.8178, 5.}	{{1.99485, 17.8178, 5.}, {1.99485, 17.8178, 5.}}	{7.63132, 1.54534, 25.}	inf	90.	64.0104	{56.465, 70.4269}	28.8773	0.149412	newly	26.	23.38	{19., 29.}	15.3077	0.0738393	Reff	0.615332	0.714135	{0.701169, 0.72866}	16.0569	ar	754.	744.98	{720., 775.}	2.58621
